# Surface Stiffness Has No Impact on MCF-7 Sensitivity to Doxorubicin

**DOI:** 10.3390/ijms241210192

**Published:** 2023-06-15

**Authors:** Egidijus Šimoliūnas, Daiva Jasmontaitė, Algimantas Skinderskis, Ieva Rinkūnaitė, Milda Alksnė, Mantas Liudvinaitis, Daiva Baltriukienė

**Affiliations:** Department of Biological Models, Institute of Biochemistry, Life Sciences Center, Vilnius University, 10257 Vilnius, Lithuania

**Keywords:** surface stiffness, MCF-7, MAPK, doxorubicin, breast cancer, mechanobiology, drug resistance

## Abstract

Resistance to the chemotherapeutic agents in the clinical management of cancer remains a significant challenge, and the mechanical environment of cancer cells is one of the major determinants of this. Stiffening of the environment is usually associated with increased chemoresistance of cancer cells, although this process depends on the type of cancer. Breast cancer is the most frequently diagnosed cancer, and more than half a million people die from it each year worldwide. In this study, we used the most frequent (70% of diagnosed cases) breast cancer phenotype, representing the MCF-7 cell line, to investigate the influence of surface stiffness on its sensitivity to one of the most commonly used anticancer drugs—doxorubicin. We showed that the mechanical environment affected MCF-7 proliferation, adhesion, and the expression and activation of mitogen-activated protein kinases (MAPKs). Furthermore, the role of MAPKs in response to doxorubicin was dependent on surface stiffness; nevertheless, surface stiffness did not affect MCF-7 resistance to doxorubicin.

## 1. Introduction

Breast cancer is the most commonly diagnosed cancer and the second leading cause of death among women worldwide [1]. Although men are also affected to a lesser extent, the most significant risk factors are gender (women) and older age [2]. In the last two decades, clinical and experimental studies have shown that breast cancers with different histopathological and biological characteristics exhibit distinct behaviours leading to different treatment responses. Therefore, therapeutic strategies should be differentiated accordingly [3]. On this basis, breast cancer patients are systematically tested for the presence of receptors, including oestrogen receptor progesterone receptor and human epidermal growth factor receptor 2 to explore tailored treatment options with molecularly targeted therapies [4]. The MCF-7 line, derived from a malignant pleural effusion in 1973, is one of the most commonly used culture models for human breast cancer and represents the most common (70% of diagnosed cases) breast cancer phenotype [5]. The anthracycline doxorubicin isolated from *Streptomyces peucetius* var. *caesius* is a potent chemotherapeutic agent that is successfully used to treat various forms of liquid and solid tumours and is currently approved for the treatment of breast cancer [4].

The spread of tumours in surrounding normal tissues inevitably leads to the destabilisation of tissue homeostasis [6]. The development of cancer causes changes in cellular biomechanics for both the cancer and surrounding cells [7]. These changes translate concomitantly into specific biomechanical cues [8]. Tumours are known to become stiff due to fibrosis during tumour progression, which affects the biochemical and physical properties of tumour cells [6,9]. These changes may affect essential features of cancer, such as increased cell proliferation, resistance to cell death, induced angiogenesis, and increased cell migration [10,11,12]. Therefore, it is important to understand how the mechanical properties of the cell surroundings affect cancer cell fate.

In this study, we evaluated how surface stiffness affects the sensitivity of the breast cancer cell line MCF-7 to doxorubicin. As known by the authors, this has not previously been tested in biologically relevant stiffness environments. Only one study analysing other breast cancer cell line MDA-MB-231 has been published, where researchers have observed stiffness-dependent MDA-MB-231 increased resistance to doxorubicin on hard tissue stiffness corresponding surfaces [13]. Nevertheless, MDA-MB-231 accounts for only 10–15% of diagnosed breast cancers as MCF-7 has the most common phenotype (70%) [4]. Therefore, it is important to determine whether surface stiffness characteristics also influence MCF-7 sensitivity to doxorubicin.

## 2. Results

### 2.1. MCF-7 Adhesion Kinetics on Surfaces with Different Stiffnesses

First, we examined how MCF-7 cells interacted with the different stiffness surfaces.

**Figure 1:**
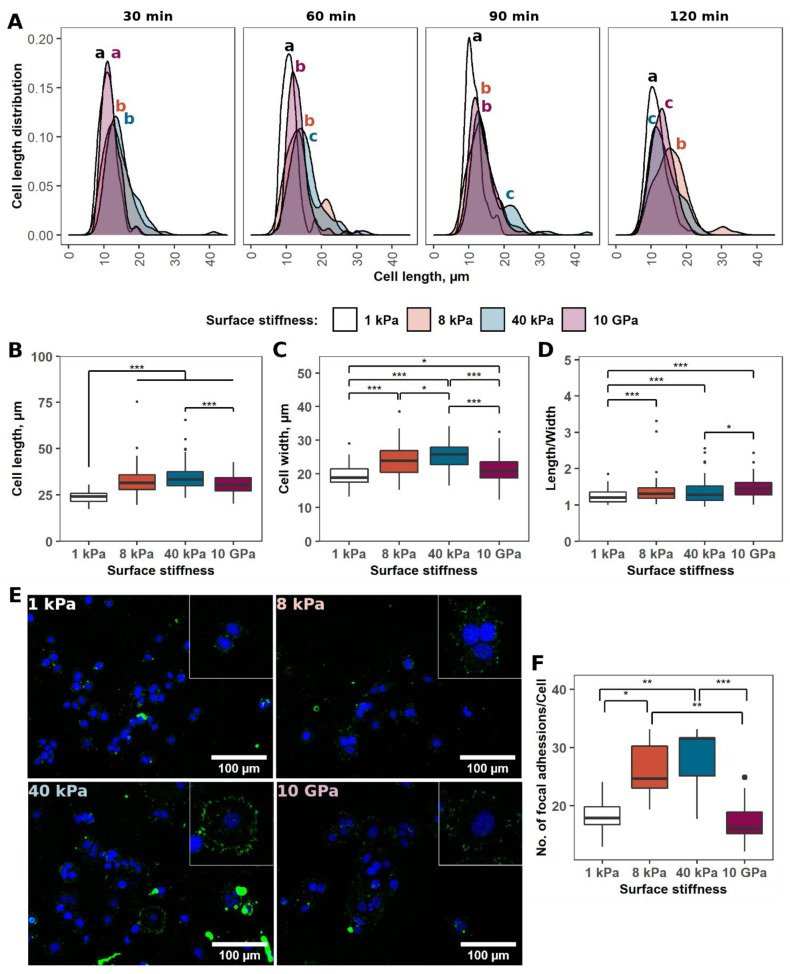
MCF-7 cell interaction with the surfaces of different stiffnesses. (**A**)—MCF-7 adhesion kinetics on surfaces of different stiffnesses. Cells were seeded onto the surfaces and their length was evaluated after 30, 60, 90, and 120 min. Correspondingly labelled letters mark significantly different groups. Two or more groups sharing the same latter had no statistically significant difference in measured MCF-7 cell length. N = 50. (**B**–**D**)—The length and width of MCF-7 cells and their ratio were measured 24 h after seeding. N = 50. (**E**)—immunohistochemistry images of focal adhesions formed by MCF-7 grown on 1, 8, 40 kPa, and 10 GPa surfaces, visualised by staining of p-FAK (Tyr397). Green—p-FAK (Tyr397), blue—MCF-7 nuclei stained with DAPI. (**F**)—quantitative evaluation of focal adhesions formed by MCF-7 grown on 1, 8, 40 kPa, and 10 GPa surfaces. N = 50. *—marks statistically significant differences between groups. *—*p* < 0.05; **—*p* < 0.01; ***—*p* < 0.001.

The cells were seeded on the tested surfaces and their adhesion was evaluated after 30, 60, 90, and 120 min (Figure 1A). The worst MCF-7 cell adhesion was observed with the softest 1 kPa stiffness hydrogel. Their size increased from 11.2 ± 2.3 µm to only 11.6 ± 2.8 µm during the two-hour evaluation. A similar tendency was observed on the other surfaces (8 and 40 kPa, and 10 GPa). However, the fastest MCF-7 cell adhesion was observed with the stiffest surfaces—40 kPa hydrogel and 10 GPa tissue culture plate surface (TCPS). After half an hour, the cells were significantly more spread on these surfaces than on hydrogels with 1 and 8 kPa stiffness. One hour after seeding, the cancer cells seeded on the 8 kPa surface caught up with the MCF-7 cells that had grown on the plastic surface. However, the cells on the 40 kPa surface continued to spread the fastest compared to MCF-7 cells seeded on the other surfaces. This trend remained constant for another 30 min. However, after two hours, the most spread cells were observed on the 8 kPa surface, while the spread of cells on the stiffest surface (40 kPa and 10 GPa) slowed somewhat. Thus, during the initial hours, the cells tended the best to adhere to the stiffest surfaces; however, later, the MCF-7 cells were most prevalent on hydrogels with average stiffness of 8 kPa. Still, the change in cell length was negligible on all tested surfaces. The average length of MCF-7 cells changed from 13.6 ± 3.7 to 15.9 ± 5 µm during 2 h on the 8 kPa stiff surface.

### 2.2. Impact of Surface Stiffness on the Morphology of MCF-7 Cells

The tested mechanical properties of the surfaces showed an impact on MCF-7 cell adhesion kinetics. Therefore, we next analysed whether surface stiffness affects breast cancer cell line morphology. MCF-7 cells were grown onto 1, 8, and 40 kPa hydrogels and 10 GPa plastic surface for 24 h, and their morphology was evaluated (Figure 1B–D). Cell length was dependent on surface stiffness (Figure 1B). The shortest MCF-7 cells were on 1 kPa hydrogels, whereas on the other surfaces, the differences between the groups were not as highlighted. Nevertheless, the longest cells after 24 h of growth were on the stiffest 40 kPa hydrogel. In contrast to cell length, MCF-7 cell width was much more affected by surface stiffness properties (Figure 1C). The cells also revealed the narrowest morphology on the softest hydrogel. As the stiffness of the hydrogel increased, the cells became wider. However, this did not fit for MCF-7 grown on plastic. In this case, the cells were wider than MCF-7 grown on 1 kPa hydrogel, but narrower than cells on the 8 kPa surface. The overall morphology also depended on the mechanical properties of the surface; however, the ratio of MCF-7 length to width changed linearly (Figure 1D). The roundest cells were on the softest 1 kPa hydrogel and the most elongated, ellipsoidal cells were on the stiffest 10 GPa plastic surface. Nevertheless, the change in cell morphology was minimal.

### 2.3. Surface Stiffness Dependent MCF-7 Adhesion

MCF-7 adhesion kinetics and morphology experiments have demonstrated that cells interact differently with surfaces of different stiffness. To confirm this observation, next we evaluated how many focal adhesions cells form on different surfaces. As for the morphology experiment, the cells were seeded onto the hydrogels at 1, 8, and 40 kPa hydrogels and onto the plastic surface of 10 GPa stiffness and allowed to interact with them for 24 h. Then, the cells were fixed and immunocytochemistry staining of p-FAK (Tyr397) was performed (Figure 1E).

The data show that MCF-7 cells form a different number of focal adhesions (Figure 1F). For hydrogels tested, the stiffer the surface, the more focal adhesions MCF-7 have formed. Cells grown on 1 kPa hydrogel formed on average 18.5 ± 3.4 focal adhesion complexes, on 8 kPa—25.9 ± 5.2, and on 40 kPa—28 ± 6. Interestingly, MCF-7 formed the same number of focal adhesions on the stiffest plastic surface as cells on the softest 1 kPa hydrogel—17.4 ± 3.9, indicating, that not only the stiffness, but also other properties of the surface have an impact on the strength of cell adhesion. Still, in the case of hydrogels, there is a linear relationship between surface stiffness and adhesion strength.

**Figure 2:**
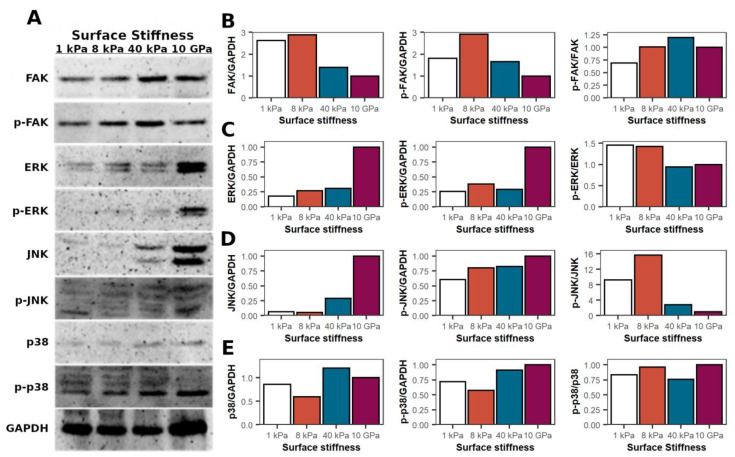
Surface stiffness impact on MCF-7 levels and phosphorylation of FAK and MAPKs. (**A**)—Western blot images of FAK and MAPKs levels of phosphorylated and total forms. The levels of kinases were evaluated 24 h after MCF-7 seeding on 1, 8, 40 kPa, and 10 GPa surfaces. GAPDH was used as a loading control. (**B**–**E**)—the expression levels of phosphorylated and total forms of FAK, ERK, JNK, and p38 were examined in MCF-7 cells using immunoblotting and densitometry. N = 1.

To better understand the differences in cell adhesion on the tested surfaces, we evaluated FAK and p-FAK (Tyr397) levels in MCF-7 for 24 h grown on hydrogels at 1, 8, 40 kPa and plastic surfaces of 10 GPa (Figure 2A). Western blot analysis showed that MCF-7, when grown on 8 kPa hydrogel, had the highest amount of FAK and p-FAK (Figure 2B). Moreover, the FAK amount decreased with increasing stiffness. The same tendency was observed for the p-FAK levels. However, on the softest 1 kPa surface, MCF-7 expressed lower levels of FAK and p-FAK compared to the cells on the 8 kPa surface, and these cells did not follow the same tendency.

### 2.4. Mitogen-Activated Protein Kinases (MAPKs) in MCF-7 When Grown in Different Stiffness Environments

Mechanical properties of the surfaces affected MCF-7 FAK kinase expression and phosphorylation. Different levels of baseline FAK may impact various cell signalling cascades in which this kinase is involved. Therefore, next, we evaluated whether MAPKs (ERK, JNK, and p38) are differently expressed and phosphorylated when MCF-7 are grown in different stiffness environments. In the case of ERK, the stiffness of the surface on which the cells grew affected its expression and phosphorylation (Figure 2A). The stiffer the surface, the more ERK was expressed and the more phosphorylated protein was detected in MCF-7 cells (Figure 2C). Interestingly, the expression of ERK was about four-fold higher on the TCPS than in the cells grown on the hydrogels. For hydrogels, the differences between the groups were not as extreme, with ERK levels increasing only slightly with increasing stiffness. In addition, the p-ERK/ERK ratio also varied between the surfaces. MCF-7 on the softest and intermediate stiffness hydrogel had a higher amount of the phosphorylated form of ERK compared to the cells grown on stiffer surfaces.

JNK kinase had a similar expression profile in MCF-7 cells grown on different stiffness surfaces (Figure 2A). The highest level of the kinase was observed in cells grown on the stiffest 10 GPa plastic (Figure 2D). Almost four-fold less JNK was observed in cells grown on the stiffest 40 kPa hydrogel. JNK levels were more than 10-fold lower on the intermediate 8 kPa and softest 1 kPa hydrogels. Nevertheless, the changes in the phosphorylated form of JNK were not as pronounced. MCF-7 grown on plastic had the greatest amount of p-JNK, and with decreasing stiffness, their levels decreased. Interestingly, the ratio of p-JNK/JNK was significantly higher in cells grown on hydrogels than in MCF-7 cells grown on plastic. The most striking result was obtained on the cells grown on hydrogels of intermediate stiffness, 8 kPa. The p-JNK/JNK ratio was almost two times lower in cells grown on the softest surface than in cells grown on the stiffest hydrogel at 40 kPa, and about four times lower than in MCF-7 cells grown on the 8 kPa surface.

The expression of p38 MAPK changed only slightly in MCF-7 cells (Figure 2A,E). However, the phosphorylated form of p38 was actively modified in MCF-7 cells grown on all surfaces. The ratio of p-p38/p38 also varied only slightly between groups.

Thus, the expression and phosphorylation of ERK and JNK in MCF-7 cells were dependent on the stiffness of the surface stiffness. The stiffer the surface, the more of these kinases were detected. However, in the case of p38, there was no clear relationship between the stiffness of the surface on which MCF-7 were grown and the expression and phosphorylation of p38 MAPK.

### 2.5. MCF-7 Proliferation on the Different Stiffness Surfaces

**Figure 3:**
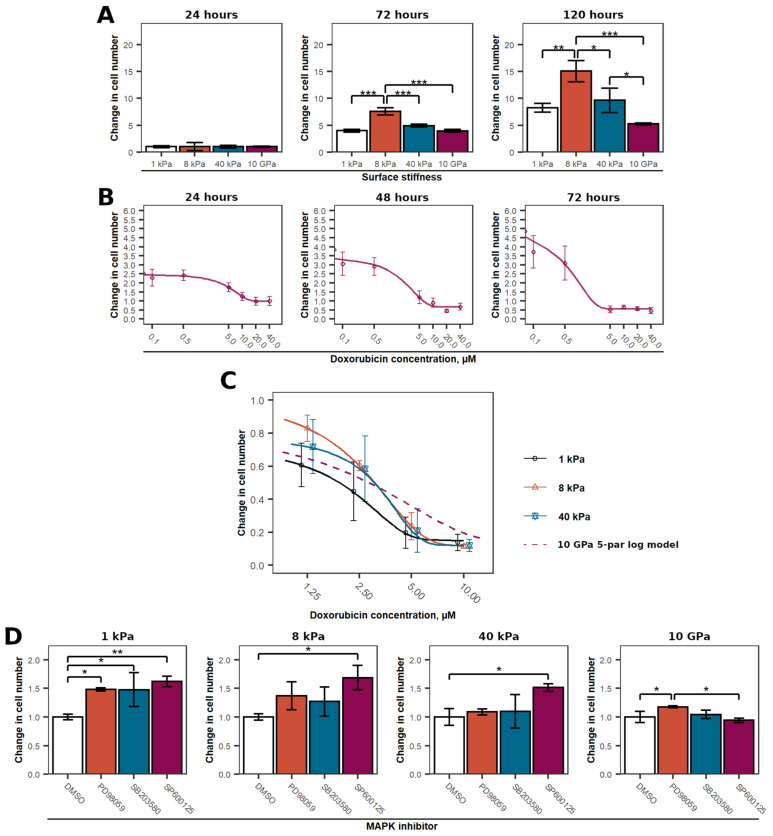
Different stiffness surfaces impact MCF-7 proliferation and sensitivity to doxorubicin. (**A**)—MCF-7 proliferation assessment on 1, 8, 40 kPa, and 10 GPa surfaces 24, 72, and 120 h after seeding. N = 3. (**B**)—Time-dependent doxorubicin cytotoxic activity to MCF-7 grown on plastic (10 GPa) tissue culture plate surface. Change in cell number due to exposure to 0.1; 0.5; 5; 10; 20; and 40 µM doxorubicin after 24, 48, and 72 h of exposure. N = 3. (**C**)—Surface stiffness impact on MCF-7 sensitivity to 1.25; 2.5; 5; and 10 µM doxorubicin concentrations 48 h after seeding. Points represent group average ± SD. Lines—5 parameter logistic models of MCF-7 response to doxorubicin treatment then grown on, 8, 40 kPa surfaces. Dash line—5 parameter logistic models of MCF-7 response to doxorubicin treatment then grown 10 GPa surface (data from (**B**) 48 h graph). N = 3. (**D**)—the role of ERK, p38, and JNK MAPK in MCF-7 cells grown on 1, 8, 40 kPa, and 10 GPa surfaces. PD98059, SB203580, and SP600125 are MEK (ERK kinase), p38, and JNK inhibitors. DMSO was used as a solvent control. N = 5. *—marks statistically significant differences between groups. *—*p* < 0.05; **—*p* < 0.01; ***—*p* < 0.001.

As we have shown above, FAK and MAP kinases in MCF-7 cells were affected by surface stiffness. Since these kinases are responsible for the regulation of cell fate, next we evaluated whether the cell proliferation rate was also affected by surface stiffness. MCF-7 was seeded on the four surfaces tested, and its proliferation was evaluated for 1, 3, and 5 days post-seeding (Figure 3A). Even after 3 days, MCF-7 grown on a hydrogel with a stiffness of 8 kPa showed higher proliferation potential compared to the cells grown on other surfaces. This was even more apparent after 5 days. Furthermore, MCF-7 on 40 kPa hydrogel was more proliferatively active than the cells grown on 1 kPa and 10 GPa surfaces. Thus, it seems that MCF-7 cells have an optimal surface stiffness requirement, on which their proliferation potential is the highest, and that deviation from this decreases the proliferation potential of the cells. In our case, MCF-7 proliferated the best on the hydrogel with stiffness of 8 kPa, which corresponds to the stiffness of breast cancer tissue.

### 2.6. Impact of the Surface Stiffness on MCF-7 Sensitivity to Doxorubicin

Finally, we evaluated whether the sensitivity of breast cancer cells to doxorubicin depends on surface stiffness. First, we tested how the cells respond to various concentrations of doxorubicin when grown on the plastic surface. Cell viability was monitored after 24, 48, and 72 h of exposure (Figure 3B).

After 24 h, none of the doxorubicin concentrations used induced cell death. Nevertheless, doxorubicin concentration lower than 10 µM tended to proliferate and increase in number. The cells began to die after an additional 24 h. This was also the case after another 24 (72 h post-treatment) hours. After 48 h, concentration higher than 10 µM doxorubicin induced cell death, and after 72 h—higher than 5 µM.

Following these results, MCF-7 grown on 1, 8, 40 kPa hydrogels were treated with doxorubicin at concentrations of 1.25, 2.5, 5, and 10 µM and their effects were evaluated after 48 h of exposure (Figure 3C). The data obtained show that MCF-7 cells were more sensitive on all hydrogels tested that MCF-7 cells grown on plastic. However, this effect was observed only after treatment with higher concentrations of doxorubicin. Beyond that, there were no pronounced differences between the hydrogels. Nevertheless, cells grown on the 8 kPa surface tended to be more resistant to the cytotoxic effect of doxorubicin when the drug concentration was below 5 µM.

### 2.7. The Role of MAPKs in MCF-7 Grown on Different Stiffness Surfaces in Response to Doxorubicin

MAPKs have been differently expressed and phosphorylated on different stiffness surfaces. Therefore, we next evaluated whether different expression and phosphorylation profiles of ERK, JNK and p38 MAPK play different roles in response to doxorubicin depending on the stiffness of the surface on which MCF-7 cells are grown. Cells were seeded on surfaces with stiffness of 1, 8, 40 kPa, and 10 GPa stiffness surfaces. At 24 h after seeding, cells were treated with specific MAPK inhibitors 30 min prior to treatment with 2.5 µM doxorubicin and the effects on MCF-7 viability were examined after additional 48 h (Figure 3D).

In MCF-7 cells grown on the softest surface, inhibition of MAPK increased their resistance to doxorubicin by at least 50%. This suggests that all of these kinases are involved in MCF-7 death signalling when cells are grown in a soft environment. On an intermediate stiffness surface, only cells with inhibited JNK kinase were significantly more resistant to doxorubicin exposure, whereas MCF-7 with inhibited ERK and p38 MAPK showed only a tendency towards higher resistance to the cytotoxic effects of doxorubicin, but the results were not statistically significant. With increasing stiffness, the influence of ERK and p38 kinase on the initiation of cell death decreased. Then MCF-7 were grown on the stiffest hydrogel at 40 kPa, only JNK was involved in the initiation of cell death. Interestingly, on the stiffest 10 GPa plastic surface on which MCF-7 grew, only ERK kinase was involved in cell death processes. Moreover, on all surfaces tested, none of the MAPK was involved in cell survival signalling. Consequently, on all surfaces relevant to tissue stiffness, only the JNK kinase played a role in cell death signalling. As stiffness decreased, p38 and ERK kinases also became relevant and were involved in cell death initiation signalling. In contrast, on an artificial plastic surface, only the ERK kinase played a role in the response to doxorubicin treatment and was responsible for regulating the MCF-7 cell death processes.

## 3. Discussion

In 2020, more than 19.3 million new cancer diagnoses and more than 10 million cancer deaths were reported worldwide. Breast cancer was the most commonly diagnosed cancer (2.3 million cases), followed by lung cancer (2.2 million cases) and prostate cancer (1.4 million cases). Lung cancer has taken the most lives (1.8 million deaths), followed by liver cancer (0.8 million deaths) and stomach cancer (0.7 million deaths) [14]. In the USA, for example, only cardiovascular diseases caused more deaths than cancer in 2020 [15]. Thus, cancer remains one of the leading causes of death in the world. Although medicine has improved significantly in this millennium, there is still no single cure for cancer. In recent years, mechanobiology has attracted much attention in the field of cancer research [16]. The data obtained show that mechanical properties of the extracellular matrix and tissue affect various cellular processes. The mechanical environment is known to affect cancer cell stiffness, migration, polarisation, death and survival, cytoskeletal and organelle organisation, and even gene expression [16]. Therefore, protein complexes involved in mechanotransduction and mechanosignalling, e.g., Polycystin-1 and 2, YAP, and TAZ, are promising therapeutic targets for cancer treatment [17]. In this study, we aimed to understand how breast cancer cell line MCF-7 behaves on soft, intermediate, and hard tissues matching stiffness surfaces. In addition, we investigated whether sensitivity to one of the most widely used anticancer drugs—doxorubicin—is affected by the mechanical properties of the surface on which MCF-7 cells grow.

The mechanical environment had an Impact on the MCF-7 cell line. Even relatively short cultivation on softer surfaces (compared to plastic) had an impact on cell adhesion, morphology, and even proliferation. A similar effect of surface stiffness has been demonstrated in other cancer cells using various methods to generate mechanical stimuli, including surface topology modifications, microfluidic systems, etc. [18,19,20,21,22,23]. These processes have been shown to be regulated by various signalling pathways, including Rho-ROCK-myosin and YAP/TAZ signalling cascades [22,24].

We also observed that surface stiffness properties influenced MAPK expression and phosphorylation. Moreover, the levels of FAK and its phosphorylated form correlated directly with increasing surface stiffness. FAK, in turn, is directly involved in the formation of the focal adhesion complex, and because MCF-7 formed more focal adhesions on stiffer hydrogels, the increased FAK levels can be explained. It is known that the FAK signalling pathway can directly activate MAPK. Thus, this is one of the mechanisms that could explain the different levels of ERK, JNK, and p38 MAPK in different stiffness environments [25].

Although surface stiffness affected various processes in MCF-7 cells, this had a minimal effect on MCF-7 sensitivity to doxorubicin. It was only observed at higher doxorubicin concentrations, whereas cells responded in the same way at lower concentrations. Nevertheless, Qin et al. recently published conflicting results [13]. The authors grew other breast cancer line MDA-MB-231 cells on polyacrylamide hydrogels with similar stiffness (10, 38, and 57 kPa) and found that a surface with 38 kPa stiffness almost doubled cell resistance to the cytotoxic doxorubicin effect, while a softer surface at 10 kPa and a stiffer one at 57 kPa had no apparent effect on cell sensitivity. In the latter case, MDA-MB-231-7 cells grown on a 10 kPa surface demonstrated very similar cytotoxicity to doxorubicin as cells grown on an 8 kPa surface in our study, whereas their results observed on a 38 kPa surface were very different from our results seen on hydrogels with 40 kPa stiffness. Thus, their published results conflict with ours, or a difference of only 2 kPa in a surface stiffness might have this tremendous effect, although this is unlikely. MDA-MB-231 cells are triple negative, whereas MCF-7 has progesterone and oestrogen receptors. In addition, MDA-MB-231 is known to be highly invasive and metastatic compared to the MCF-7 breast cancer line, thus these cells might be more sensitive to the mechanical stimuli. Continuing, Joyce et al. performed a similar study using soft alginate hydrogels (0.2 and 2 kPa) [26]. Their results are in line with our data obtained with the softest hydrogel (1 kPa), further supporting our study.

MAPKs are responsible for the regulation of various cellular processes, and in particular, they are important in cell responses to various stressors [27]. The different levels of the total and phosphorylated forms of MAPK may have implications for their role in response to the anticancer drug doxorubicin. We confirmed that ERK, JNK, and p38 play different roles in the response of a cell to the different stiffness of the microenvironment. JNK kinase was responsible for cell death signalling on all biologically relevant stiffness surfaces, whereas p38 kinase was involved in cell death signalling only on the softest surface and its role rapidly decreased with increasing stiffness. Interestingly, the ERK kinase was only responsible for cell death signalling in MCF-7 grown on the softest 1 kPa hydrogel and a much stiffer artificial 10 GPa plastic surface. This shows the importance of conducting research in an environment that corresponds to the real situation in order to draw relevant conclusions [26].

In summary, mechanical environment has an impact on various oestrogen and progesterone receptors positive breast cancer cell line MCF-7 processes, including adhesion kinetics, focal adhesion complex formation, proliferation, and even FAK and MAPKs expression and phosphorylation profiles. Nevertheless, surface mechanical stiffness had a negligible effect on MCF-7 sensitivity to the anticancer drug doxorubicin. Only a slight change in MCF-7 cell resistance was observed on stiffer surfaces after exposure to larger drug concentrations. Thus, it may be that the cytotoxic effect of doxorubicin in the MCF-7 cells is not mediated throughout mechanosignalling pathways. Nevertheless, ERK, JNK, and p38 MAPK in MCF-7 cells played different roles in response to doxorubicin in surroundings of different stiffness. As different anticancer drugs have various mechanisms of action, their effectiveness to MCF-7 in different stiffness environments might be different from doxorubicin. Therefore, more research is needed to understand why different cancer cells respond differently to anticancer drugs in different stiffness environments and what the mechanism of this phenomenon is.

## 4. Materials and Methods

### 4.1. Cell Culture

The commercially available breast cancer cell line MCF-7 was used for the experiments (Cell line Service GmbH; Eppelheim, Germany). Cells were grown in Dulbecco’s Modified Eagle Medium (Thermo Fisher Scientific Inc., Waltham, MA, USA) supplemented with 10% fetal bovine serum (FBS) (Thermo Fisher Scientific Inc.) and 1% streptavidin/penicillin (Thermo Fisher Scientific Inc.) in the incubator with 95% humidity and 5% CO_2_ concentration at 37 °C. Cells were divided into new flasks after monolayers reached 70–80% confluence. Cells were dissociated from the surface of the tissue culture flasks using a 0.05% trypsin/EDTA (Thermo Fisher Scientific Inc.; Merck KGaA, Darmstadt, Germany respectively) solution. 

### 4.2. Preparation of Acrylamide Hydrogels with Different Stiffnesses

Three acrylamide hydrogels of different stiffnesses—1, 8, and 40 kPa stiffness—were used in this study. They were prepared according to the protocol of Tse and Engler [28]. Briefly, polyacrylamide hydrogels were attached to glass coverslips. Two types of coverslips were used—round and square 22 mm coverslips. Round coverslips were treated with 2% 3-aminopropyltrimethoxysilane (APTES) (Merck KGaA), prepared in acetone to make their surface hydrophilic and increase their binding to the polyacrylamide. Square coverslips were coated with dichloromethylsilane (CMS) (Merck KGaA) to make the glass surface hydrophobic and to reduce its binding to the polyacrylamide. Acrylamide (AA) (Merck KGaA) and bis-acrylamide (BIS) (Merck KGaA) mixtures of 35 μL in volume were prepared: 40 kPa–8% AA, 0.48% BIS, 8 kPa–5% AA, 0.225% BIS, and 1 kPa–5% AA, 0.03% BIS after mixing with 1/1000 volume of tetramethylethylenediamine (Bio-Rad Laboratories, Inc., Hercules, CA, USA) and 1/100 volume of 10 % ammonium persulfate (Merck KGaA), were pipetted onto round glass coverslips and covered with square coverslips. After one hour of incubation in a humid environment at 37 °C, the square coverslips were separated from the round ones containing the synthesised polyacrylamide hydrogels. The gels were then washed 3 times for 5 minutes with phosphate buffer saline (PBS) (Thermo Fisher Scientific Inc.) to remove monomer residues. The gels were then coated with type I collagen using the protein cross-linker Sulfo-SANPAH (Thermo Fisher Scientific Inc.). The elasticity of the substrate was confirmed by atomic force microscopy measurements (Table S1, Figure S1).

### 4.3. Kinetics of Cell Adhesion

A suspension of 3 × 10^4^ cells/mL was seeded on four different stiffness surfaces—1, 8, and 40 kPa polyacrylamide hydrogels and 10 GPa plastic surface. After seeding, samples were placed in an incubator with 95% humidity, 5% CO_2_ concentration, and 37 °C temperature. At 30, 60, 90, and 120 min after seeding, cells were observed by bright-field microscopy and 2D cell morphology was captured with a CCD camera (QImaging EXi Blue CCD). Cell length was measured using the ImageJ (ImageJ 1.8.0_112) image processing program.

### 4.4. Assessment of Cell Morphology

Cell morphology was evaluated by F-actin staining as described previously [29]. MCF-7 cells were grown on four different stiffness surfaces—1, 8, and 40 kPa polyacrylamide hydrogels and 10 GPa plastic surface—for 24 h. The samples were fixed with 4% paraformaldehyde (Merck KGaA) at room temperature (RT) for 15 min with gentle agitation at 25 rpm. Then, samples were rinsed 3x for 5 min with 0.2% Triton X-100 (Merck KGaA) in PBS and stained with 5 U/mL rhodamine-phalloidin and 4 µg/mL 4’,6-diamidino-2-phenylindole (DAPI) (Thermo Fisher Scientific Inc.) working solution in PBS in the dark for 60 min at RT. Samples were then visualised using an inverted fluorescence microscope (Olympus IX51; Olympus Corporation, Shinjuku, Tokyo, Japan). Differences in cell morphology and shape were evaluated by measuring cell width, length, and aspect ratio (the ratio between width and length) using the ImageJ (ImageJ 1.8.0_112) image processing program.

### 4.5. Immunocytochemistry

Cells were seeded at a density of 3 × 10^4^ cells/mL onto four different stiffness surfaces—1, 8, and 40 kPa hydrogels and a control 10 GPa plastic surface. After 24 h, the cells were fixed with 4% formaldehyde in PBS at RT for 10 min with gentle agitation (25 rpm). Samples were then rinsed twice with 0.05% Tween-20 (Merck KGaA) in PBS, permeabilised with 0.2% Triton X-100 in PBS at RT for 5 min, and cells were blocked with 3% BSA (Merck KGaA) and 10% FBS in PBS for 30 min. Next, cells were incubated with primary antibodies against phospho-FAK (Tyr397) (p-FAK) (Cell Signaling Technology, Inc., Danvers, MA, USA, Cat. No. 3283) protein. Antibodies were prepared in a blocking solution and cells were incubated for 1 h at RT. Then, samples were washed 3x for 5 min with 0.05% Tween-20 solution and incubated with secondary goat anti-rabbit Alexa Fluor 488 (Thermo Fisher Scientific, Inc., Cat. No. A-11008)—conjugated antibodies prepared in blocking solution in the dark for 1 h at RT. Samples were then washed 3x for 5 min with PBS at RT. In addition, cells were stained with 4 µg/mL (DAPI) prepared in PBS for 5 min in the dark at RT. Finally, samples were washed 3x for 5 min with PBS and visualised with a fluorescence microscope (Olympus IX51). The program ImageJ (1.8.0_112) was used for image processing.

### 4.6. Evaluation of Focal Adhesions

The strength of cell adhesion on the tested scaffolds was quantitatively assessed by counting the focal adhesions formed on the surfaces of different stiffness within the cells 24 h after seeding. Images of p-FAK (Tyr397) (Cell Signaling Technology, Inc., Cat. No. 3283) obtained by immunocytochemistry were used for analysis. Focal adhesions were calculated according to the protocol of Horzum et al. [30]. ImageJ (1.8.0_112) image processing software was used for analysis.

### 4.7. Cell Proliferation

MCF-7 were seeded onto four different surfaces with a stiffness of 1, 8, and 40 kPa, and 10 GPa at a density of 2 × 10^4^ cells/gel or well. Cell numbers were determined 24, 48, 72, and 96 h after seeding using the 3-(4,5-dimethylthiazol-2-yl)-2,5-diphenyltetrazolium bromide (MTT) (Merck KGaA) assay. For incubation, 0.2 mg/mL MTT solution was used for 1 h at 37 °C. The formed formazan crystals were dissolved with DMSO (Merck KGaA) and the solution absorbance was measured at 570 nm using Varioscan Flash (Thermo Fisher Scientific, Inc.) plate reader. Data were standardised according to each group 24 h MTT absorption results. Cell doubling times were calculated from cell counts at 48, 72, and 96 h using the following equations:

(1) $$Growth\;rate=\frac{ln(N(t)/N(0))}{t};$$

(2) $$Doubling\;time=\frac{ln(2)}{Growth\;rate}.$$

where *N*(*t*)—the number of cells at time *t*; *N*(0)—the number of cells at time 0; *t*—time in hours.

### 4.8. Evaluation of the Cytotoxicity of Doxorubicin

MCF-7 cells were seeded on the tested surface at a density of 6400 cells/cm^2^. The next day, the growth media were replaced with media containing different concentrations of doxorubicin (Merck KGaA)—from 0.05 to 40 µM. The change in cell number was evaluated after 24, 48, and 72 h using the MTT method. For incubation, 0.2 mg/mL MTT solution was used for 1 h at 37 °C. The formed formazan crystals were dissolved with DMSO and the solution absorption was measured at 570 nm with Varioscan Flash plate reader. The relative change in cell number was assessed in two ways, compared to the initial cell number before treatment with doxorubicin and compared to the untreated control at the respective time points.

### 4.9. The Evaluation of the Role of MAP Kinases

To determine the role of MAP kinases in MCF-7 during doxorubicin exposure, their specific inhibitors were used. ERK kinase was inactivated by inhibiting its kinase MEK1 with PD98059 (Merck KGaA). JNK and p38 were directly inhibited with SP600125 (Merck KGaA) and SB203580 (Merck KGaA), respectively. MCF-7 was seeded on four different stiffness surfaces at a concentration of 4 × 10^4^ cells/surface. The next day, MCF-7 was treated with 40 µM SP600125, 30 µM SB203580, and 0.4 µM PD98059 30 min before treatment with 2.5 µM of doxorubicin. The control group was treated with the solvent DMSO. MCF-7 cell viability was evaluated after 48 h, and MTT assay was performed for two hours. The formed formazan crystals were dissolved with DMSO, and the solution absorptions were measured at 570 nm with Varioscan Flash plate reader. Data were standardised according to the cell number of the control group treated with DMSO control group for the cell number of each surface stiffness.

### 4.10. Western Blot

To assess the expression and phosphorylation of MAP kinases and their primary targets c-Jun and c-Fos, MCF-7 was seeded at a density of 7 × 10^5^ cells/dish on standard glass Petri dishes coated with polyacrylamide hydrogels with stiffnesses of 1, 8, and 40 kPa and on a control plastic surface (10 GPa stiffness). After incubating the cells for 24 hours, they were lysed with ice-cold lysis buffer (10 mM Tris HCl, pH 7.4, 50 mM NaCl (Merck KGaA), 5 mM EDTA, 50 mM NaF (Merck KGaA), 1% Triton X-100) and supplemented with protease and phosphatase inhibitors: aprotinin (1 mg/mL) (Merck KGaA), PMSF (1 mM) (Merck KGaA), and Na_3_VO_4_ (1 mM) (Merck KGaA). Protein concentration was estimated using the Bradford assay. Equal amounts of protein were separated by SDS-PAGE on 12% polyacrylamide gels and transferred to a nitrocellulose membrane (semi-dry transfer, 10% methanol). Membranes were then blocked with 5% BSA dissolved in TBST (5 mM Tris HCl pH 7.5, 0.1% Tween 20, 154 mM NaCl) for 1 h at RT. Primary antibodies (Table 1) were diluted in 5% blocking solution according to the manufacturer’s recommendations and applied to the membranes overnight at 4 °C. After incubation, the membranes were washed 3x for 3–5 min in TBST and then incubated with the secondary antibodies (Gt anti-MS Fc (Thermo Fisher Scientific, Inc.; 31430), Gt anti-Rb Fc (Thermo Fisher Scientific, Inc.; 65-6120)) diluted in blocking buffers according to the manufacturer’s recommendations for 1 h at RT. Again, the membranes were washed 3x for 3–5 min in TBST and incubated for 3 min with Pierce ECL Western Blotting Substrate. Protein expression was visualised with ChemiDoc XRS+ Molecular Imager (Bio-Rad Laboratories, Inc.) using Image Lab Software (version 3.0.1). Western blot images were analysed using the ImageJ (1.8.0_112) program. GAPDH protein was used as a loading control. The amounts of phosphorylated proteins were normalised to that of the respective total protein.

### 4.11. Statistical Analysis

All statistical analyses were performed using R program package (RStudio 4.0.3). Data are presented as median ± interquartile region or mean ± standard deviation (SD) of at least 3 independent experiments with at least 3 samples per group, except the Western blot analysis. The Shapiro–Wilk test was used to determine the normality of the data (when *n* ≥ 5), and in the case of *n* < 5, a normal distribution of the data was assumed. All data were normally distributed, thus statistically significant differences between the two groups were determined using the *t*-test; for three or more groups, a one-way analysis of variance (ANOVA) was performed; and the Tukey post hoc test was used to assess statistically significant data differences. *p*-values with <0.05 were considered statistically significant. The ggplot2 library was used for graph preparation and statistically significant differences in graphs were marked with * signs. *—*p* < 0.05, **—*p* < 0.01 and ***—*p* < 0.001, or if other symbols were used, the markings below the graph are explained.

## Figures and Tables

**Table 1:** The list of primary antibodies used.

Protein	Manufacturer	Catalogue No.
FAK	Cell Signaling Technology, Inc.	3285
Phosho-FAK	Cell Signaling Technology, Inc.	3283
ERK	Cell Signaling Technology, Inc.	137F5
Phospho-ERK	New England Biolabs, Inc. (Ipswich, MA, USA)	9109
JNK	Cell Signaling Technology, Inc.	56G8
Phospho-JNK	Cell Signaling Technology, Inc.	9251L
p38	Thermo Fisher Scientific, Inc.	331300
Phospho-p38	Thermo Fisher Scientific, Inc.	MA5-15218
GAPDH	Santa Cruz Biotechnology, Inc. (Dallas, TX, USA)	SC-365062

## Data Availability

The data that support the findings of this study are available from the corresponding author, E.Š., upon reasonable request.

## Supplementary Materials

The supporting information can be downloaded at: https://www.mdpi.com/article/10.3390/ijms241210192/s1.
