# Supplementary material for: Surface Stiffness Has No Impact on MCF-7 Sensitivity to Doxorubicin
Source: Int J Mol Sci. 2023 Jun 15;24(12):10192. doi: 10.3390/ijms241210192 (PMC10299124; doi:10.3390/ijms241210192)
Supplement: Supplementary file 1 [file ijms-24-10192-s001.zip › ijms-2445519-supplementary.pdf]

Supplementary Materials

Table S1. Calculated hydrogel stiffness values from atomic force microscopy measurements. Data represented as mean ± SD.

Type of poly-acrylamide hydrogel	Softview 1 kPa standard	Softview 8 kPa standard	Softview 50 kPa standard	1 kPa gel (Soft sample)	8 kPa gel (Intermediate sample)	40 kPa gel (Stiff sample)
**Hydrogel stiffness measured with AFM**	1.14±0.69	8.53±2.84	48.41±7.22	1.08±0.55	9.20±1.87	37.65±5.47
**Number of measurements**	14	23	12	39	47	53

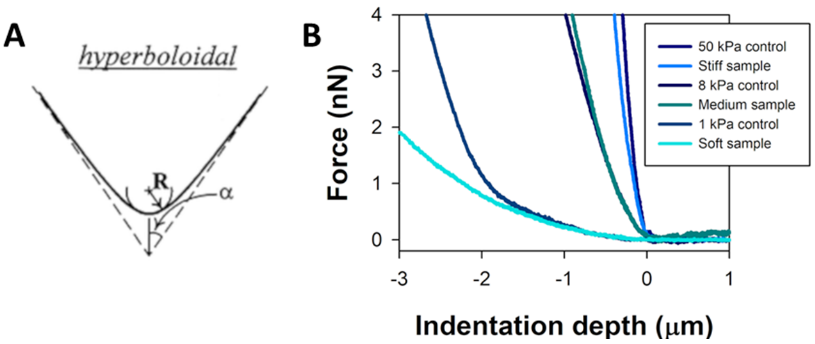

Figure S1. (**A**) Scheme of a hyperboloidal AFM tip used in the analysis: α, semivertical angle; R, radius of curvature of the tip apex. (**B**) Representative force-indentation curves of each tested sample.
